# A Comparison of a Drug Coated Balloon With Drug Eluting Stent Strategy for Treating Coronary Bifurcation Lesions

**DOI:** 10.1002/ccd.70273

**Published:** 2025-10-28

**Authors:** Natasha Corballis, Ioannis Merinopoulos, U. Bhalraam, Tharusha Gunawardena, Vasiliki Tsampasian, Rajkumar Natarajan, Upul Wickramarachchi, Mohamed Mohamed, Allan Clark, Mamas A. Mamas, Vassilios S. Vassiliou, Simon Eccleshall, SPARTAN‐Norfolk Consortium, Tim Gilbert, Clint Maart, Johannes Reinhold, Paul Richardson, Alisdair Ryding, Trevor Wistow, Chris Sawh, Sulfi Sreekumar

**Affiliations:** ^1^ Department of Medicine University of East Anglia Norwich UK; ^2^ Department of Cardiology Norfolk and Norwich University Hospital Norwich UK; ^3^ Institute of Health Informatics University College London London UK; ^4^ Keele Cardiovascular Research Group, Centre for Prognosis Research, Institute for Primary Care and Health Sciences Keele University Keele UK

**Keywords:** complex PCI, coronary bifurcation lesions, drug coated balloons, PCI

## Abstract

**Background:**

The treatment of coronary bifurcation lesions (CBL) remains complex and associated with a higher rate of long‐term adverse cardiovascular events due to anatomical and procedural complexity.

**Aims:**

We compared procedural outcomes between a drug coated balloon (DCB) only approach and a 2nd generation drug eluting stent (DES) for treating de novo CBLs.

**Methods:**

We retrospectively identified all patients with CBL treated with either a DCB only or DES only strategy, including all coronary bifurcations and compared a bifurcation‐oriented composite endpoint (BOCE) of cardiovascular death, target bifurcation‐related myocardial infarction (TB‐MI), and clinically driven target bifurcation revascularization (TBR) using nationally obtained clinical outcome measures from 2015 to 2019. A propensity score matched analysis was undertaken.

**Results:**

Of 2113 patients, 1030 patients were treated with a DCB and 1083 with a DES. There was higher lesion complexity in the DCB group, and propensity score‐matched analysis was utilized. This included a total of 2052 patients (1026 in each arm). The median age was 68 (59−75), and all clinical presentations were included. The median follow‐up time was 3.6 (2.5−4.8) years with 501 (48.8%) patients having follow‐up available at 5 years. Propensity matched analysis showed a significant increase in events (14.0% vs. 9.9% respectively) (HR: 1.39 [1.08−1.79], *p* = 0.01) when using DES compared to DCB, driven predominantly by an increase in TBR (8.9% vs. 5.0%) (HR: 1.79 [1.27−2.50], *p* ≤ 0.001) and TB‐MI (3.0% vs. 1.6%) (HR: 1.92 [1.05−3.57], *p* 0.03).

**Conclusions:**

The use of DCB‐only in a coronary bifurcation is a safe alternative in treating CBL, within the limitations of a retrospective single center analysis. An appropriately designed RCT is now required.

**Abbreviations**
BOCE: bifurcation oriented composite endpoint
CBL: coronary bifurcation lesion
DCB: drug coated balloon
DES: drug eluting stent
TLR: target lesion revascularization
TL‐MI: target lesion myocardial infarction

## 1 Background

Coronary bifurcation lesions (CBL) account for at least one in five angioplasties undertaken [1] and are associated with a higher rate of peri‐procedural complications and long‐term major adverse cardiovascular events [2] than non‐bifurcation lesions. Target lesion revascularization occurs in up to 10% of bifurcation lesions at 3 year follow‐up [3, 4] and some studies show up to 70% higher rates of treated vessel failure compared to non‐bifurcation PCI [5]. A provisional main vessel single stent strategy is recommended, with the European Bifurcation Club (EBC) consensus documents advocating a provisional main vessel strategy in the majority of bifurcation lesions, even in true CBL, where the side branch lesion length is < 10 mm [6] as this strategy has been associated with lower all‐cause mortality than a two‐stent strategy [7].

A drug coated balloon (DCB) strategy has to date only been investigated in randomized controlled trials for side branch treatment [8, 9, 10, 11]. There is increasing evidence to support the safety and efficacy of a DCB approach to PCI, including complex and LMS PCI [12, 13, 14, 15, 16], and to potentially simplify a bifurcation strategy by preventing carina shift from stent implantation whilst allowing for side branch late lumen gain. We therefore sought to investigate if DCB‐only angioplasty is a safe and effective strategy for treating CBL.

## 2 Methods

This investigator‐initiated cohort study was conducted at a single‐center, high volume PCI department. All patients who undergo PCI are prospectively entered into a clinical database where all procedural details are recorded. Approval was obtained from the Northwest Haydock (17/NW/0278) UK research ethics committee and Institutional Board approval by the Norfolk and Norwich University Hospital. We interrogated our clinical database to identify all patients who underwent bifurcation PCI from 2015 to 2019. Patients with a DCB only or drug eluting stent (DES) only procedure to a CBL were identified and included in this analysis. Clinical and angiographic details were obtained from our clinical database and supplemented from Hospital Episodes Statistics (HES) data. All clinical outcomes were obtained retrospectively from two national databases of clinical outcomes, HES from NHS Digital and the National Institute of Cardiovascular Outcomes Research (NICOR). The confidentiality advisory group waived the requirement for patient consent given the retrospective nature of our study (17/CAG/0145).

CBL were defined as a significant coronary stenosis within 4 mm of a branch, with the side branch being ≥ 2 mm in diameter by visual estimation, a definition recommended by the EBC‐ARC consensus document [17]. All angiograms were reviewed independently by an expert operator (N.C./I.M.) to determine the presence of a bifurcation lesion. The Medina score was calculated for all bifurcation lesions and then classed as a true bifurcation lesion if Medina 1.1.1, 1.0.1, or 0.1.1. All DCBs used were paclitaxel (98% SeQuent Please NEO) while all DES were 2nd generation. Given the retrospective nature of the analysis, the strategy of CBL PCI was fully at the discretion of the operator. We have outlined in further detail our approach to DCB only bifurcation PCI in the discussion. The choice of DCB or DES approach for a bifurcation lesion was at the operator's discretion reflecting variations within our own practice and DCB PCI was undertaken according to consensus recommendations [18]. HES provided ICD‐10 diagnostic codes, ensuring that all hospital admissions, including those outside of our center, were identified and reported. All patients who underwent revascularization had angiographic review to determine if the revascularization was due to a target lesion.

Our primary composite endpoint was defined as: cardiovascular death, target bifurcation‐related myocardial infarction (TB‐MI), and clinically driven target bifurcation revascularization (TBR), as recommended by the European Bifurcation Club Academic Research Consortium document [17]. Any clinically apparent peri‐procedural MI was included in the definition of TL‐MI. Secondary endpoints were the individual components of the composite endpoint. All deaths were adjudicated by a committee blinded to the treatment strategy (overseen by V.S.V.), and determined to be cardiovascular or non‐cardiovascular based on the academic research consortium definition [19], using ICD‐10 codes for cause of death. TBR was defined based on the Bif‐ARC document [17], as within 5 mm from the stent edge/balloon edge in both the MV and SB. These events were adjudicated by two researchers (N.C./I.M.) and overseen by S.E. Patient frailty scores were calculated based on ICD‐10 hospitalization codes using the validated Hospital Frailty Risk Score [20].

### 2.1 Statistical Analysis

All statistical analysis was undertaken using R 4.3.1 with RStudio Server 2023.06.0 by an independent data scientist. Given the number of observations, Kolmogorov−Smirnov tests were used in tandem with visual density and Q−Q plot evaluation to determine normality. Normally distributed variables were reported as mean ± standard deviation (SD) and non‐normally distributed variables were reported as median (interquartile range [IQR]). To determine significant group differences, the appropriate independent parametric and nonparametric tests were used. The *α* significance level for all statistical tests was set at 0.05. Univariate Cox regression was undertaken for all variables against the composite end point to identify predictors. Cumulative hazard plots were created, and the log‐rank test performed to determine significant differences between the arms. Multivariate Cox‐regression models were created using all the pre‐selected variables in the model. A mixed effect Cox model was used to create multivariate models; accounting for the matched paired observations in this study. The assumptions of the Cox proportional hazard models, particularly the proportional hazards assumption, was checked with the coxzph() function from the survival package in R, performing the Schoenfeld Individual Test on the individual variables of the model, as well as a global Schoenfeld Test *p* value. Propensity score matching was done using the Matchit package for R [21]. The 1:1 ratio “genetic” matching method with replacement was utilized to match for the following predetermined variables: age, gender, clinical presentation, true bifurcation, history of MI, history of PCI, history of AF, history of DM, vessel treated, presence of heavy calcification, presence of diffuse disease, presence of tortuosity, denovo vessel diameter, denovo lesion length, and creatinine against the intervention used (DCB/DES). Overall match performance was assessed by analyzing the SMD differences both at a univariate and global level. Characteristic tables of the matched cohort were then generated for full transparency. Patients with missing data were excluded from the propensity matched analysis (8/2113 patients, 0.38%). Follow‐up was capped at 5 years.

## 3 Results

**Figure 1:**
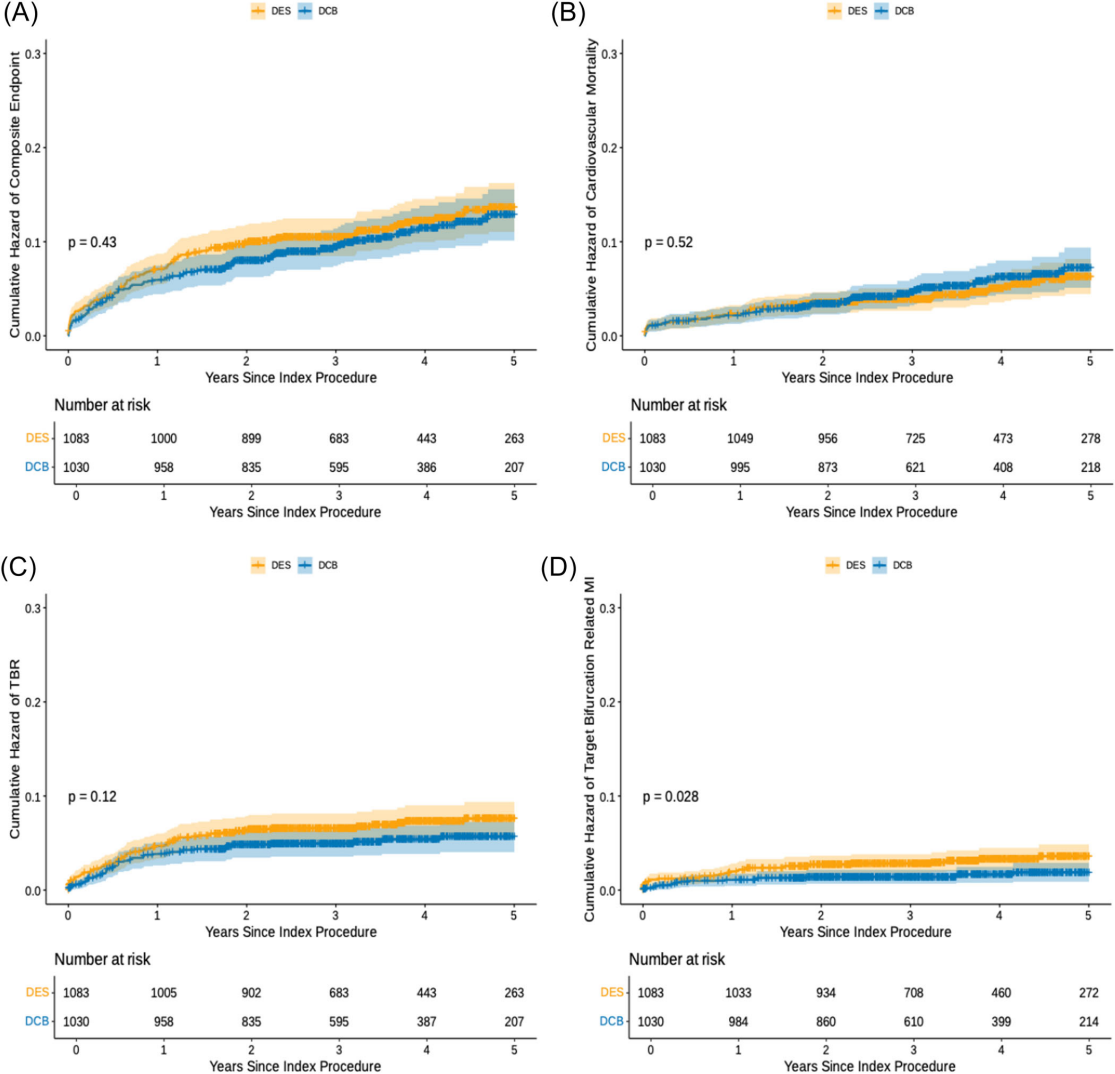
Cumulative hazard plots for the full, unmatched cohort. (A) Shows the cumulative hazard plot for the unmatched composite endpoint. (B) Shows the cumulative hazard plot for unmatched cardiovascular mortality. (C) The cumulative hazard plot for unmatched target lesion revascularization. (D) The cumulative hazard plot for unmatched target lesion myocardial infarction. [Color figure can be viewed at wileyonlinelibrary.com]

Of 2176 patients with bifurcation lesions treated between January 2015 and November 2019 with either a DCB or a DES for any clinical presentation, 61 patients opted out from HES follow‐up hence excluded. A further 51 patients with cardiogenic shock were excluded and 63 patients who were treated with both DES and DCB in the same bifurcation. A total of 2113 patients were included in this analysis, with subsequent matching of 1026 patients in the DCB cohort; with 1026 patients from the DES cohort (Supporting Information S1: Figure 1).

The baseline characteristics of the whole cohort are shown in Supporting Information S1: Table 1. Patients excluded from the matching are shown in Supporting Information S1: Table 2. The unmatched lesion characteristics (Supporting Information S1: Table 3) were significantly different between the two groups with significantly more true bifurcations in the DCB group and more lesion complexity due to calcium, tortuosity, and diffuse disease. Thus propensity matching was undertaken.

Table 1 below shows the baseline characteristics of the matched cohort. The median age was 68 years old [59–75], 19% of patients were female with no significant difference in frailty between the two groups. There were 26% of patients presenting with STEMI in both groups.

**Table 1:** Propensity matched baseline patient, lesion, and procedural characteristics.

Patient characteristics	DCB (*n* = 1026) (*n*, %)	DES (*n* = 1026) (*n*, %)	*p* value
Female sex	203 (20)	190 (19)	0.47^1^
Age (median, IQR)	68 (59−75)	68 (58−75)	0.52^2^
Frailty			0.52^3^
Low	1004 (98)	1009 (98)	
Intermediate	22 (2.1)	17 (1.7)	
High	0 (0)	0 (0)	
Hypertension	503 (49)	455 (44)	**0.03** ^1^
Dyslipidaemia	242 (24)	252 (25)	0.61^1^
Previous CVE	55 (5.4)	43 (4.2)	0.21^1^
Peripheral vascular disease	37 (3.6)	25 (2.3)	0.09^1^
Previous MI	250 (24)	251 (24)	0.80^1^
Previous PCI	260 (25)	266 (26)	0.76^1^
Previous CABG	55 (5.4)	44 (4.3)	0.21^1^
COPD	57 (5.6)	63 (6.1)	0.57^1^
Family history of coronary disease	177 (17)	164 (16)	0.44^1^
Diabetic	199 (19)	204 (20)	0.78^1^
Current/ex‐smoker	601 (59)	593 (61)	0.58^1^
AF	84 (8.2)	85 (8.3)	0.94^1^
Creatinine	83 (71‐97)	84 (72‐96)	0.63^1^
Clinical presentation			0.92^1^
Stable	376 (37)	380 (37)	
ACS (unstable angina/NSTEMI)	383 (37)	374 (36)	
STEMI	267 (26)	272 (27)	
*Lesion/procedure characteristics*			
Site of bifurcation disease			0.049^3^
LMS	49 (4.8)	44 (4.3)	
LAD	616 (60)	571 (56)	
Cx	250 (24)	317 (31)	
RCA	109 (11)	91 (8.9)	
Graft	2 (0.2)	3 (0.3)	
Bifurcation lesion characteristics			
True bifurcation	306 (30)	290 (28)	0.44^1^
Type (Medina)			0.08^3^
111	206 (20)	203 (20)	
110	173 (17)	166 (16)	
101	49 (4.8)	39 (3.8)	
011	53 (5.2)	51 (5.0)	
100	153 (15)	165 (16)	
010	243 (24)	290 (28)	
001	149 (15)	112 (11)	
Treatment strategy			0.06^3^
One vessel strategy	956 (93)	984 (95.8)	
Two vessel strategy	70 (7)	43 (4.2)	
Main vessel diameter, median (IQR)	3.0 (2.75−3.5)	3.0 (2.75−3.5)	0.465^2^
Treated side branch diameter, median (IQR)	2.7 (2.25−3)	2.7 (2.25−3)	0.66^2^
Main vessel treated length	20 (20−30)	24 (18−32)	0.47^2^
Treated side branch length	15 (15−20)	15 (12−20)	**0.001** ^**2**^
Heavy calcification	289 (28)	293 (29)	0.84^1^
Diffuse disease	329 (32)	298 (29)	0.14^1^
Tortuosity	215 (21)	216 (21)	0.96^1^
Intravascular imaging			0.27^1^
IVUS	22 (2.1)	33 (3.2)	
OCT	9 (0.9)	16 (1.6)	
Pressure wire use	118 (12)	86 (8.4)	**0.01** ^**1**^
Contrast used, median (IQR)	130 (100−160)	140 (100−170)	**< 0.001** ^3^
Fluoroscopy time (mins) median (IQR)	12 (8−17)	12 (9−20)	**0.003^3^**

*Note:* ^1^Chi‐squared test, ^2^Wilcoxon Rank Sum test, ^3^Fisher's exact test. Bold values indicate Statistically significant at *p* < 0.05.

Abbreviations: ACS = acute coronary syndrome, CABG = coronary artery bypass grafting, COPD = chronic obstructive pulmonary disease, CVE = cerebrovascular event, Cx = circumflex artery, LAD = left anterior descending artery, LMS = left main stem, MI = myocardial infarction, PCI = percutaneous coronary intervention, RCA = right coronary artery.

The propensity matched lesion characteristics are shown below (Table 1). There were slightly more LMS treated in the DCB group than the DES group (4.8% vs. 4.3%). A one vessel/provisional treatment strategy was adopted in 93% of DCB patients compared to 96% of DES patients. There was a significant reduction in the procedure duration (fluoroscopy time: 12 [8–17] min compared to 12 [9–20] min, *p* = 0.003) in the DCB group.

Median (IQR) follow‐up for all patients was 3.4 (2.5–4.8) years for DCB and 3.7 (2.5–4.8) years for DES with 501 (48.8%) patients having follow‐up available at 5 years.

The unmatched analysis showed no significant difference in the composite endpoint between the two groups, with full results shown below (Figure 1, Panel A–D and Table 2).

**Table 2:** Univariate outcomes for both the unmatched and matched cohorts with the composite endpoint and individual components of the composite.

Outcome	Unmatched DCB *n* (%) 1030	DES *n* (%) 1083	HR (95% CI)	*p* value	Propensity matched DCB *n* (%) 1026	DES *n* (%) 1026	HR (95% CI)	*p* value
Composite endpoint	102 (9.9)	125 (12.0)	1.14 (0.88−1.48	0.33	102 (9.9)	142 (14)	HR: 1.39 (1.08−1.79	**0.01**
TBR	51 (5.0)	74 (6.8)	1.36 (0.95−1.95)	0.088	51 (5.0)	91 (8.9)	1.79 (1.27−2.50)	**< 0.001**
CD	54 (5.2)	54 (5.0)	0.91 (0.62−1.32)	0.61	54 (5.3)	54 (5.3)	0.94 (0.65−1.37)	0.75
TB‐MI	16 (1.6)	33 (3.0)	1.93 (1.06−3.51)	**0.028**	16 (1.6)	31 (3.0)	1.92 (1.05−3.57)	**0.03**

*Note:* Bold values indicate Statistically significant at *p* < 0.05.

Abbreviations: CD = cardiovascular death, CI = 95% confidence intervals, TB‐MI = target bifurcation‐related myocardial infarction, TBR = target bifurcation revascularization.

**Figure 2:**
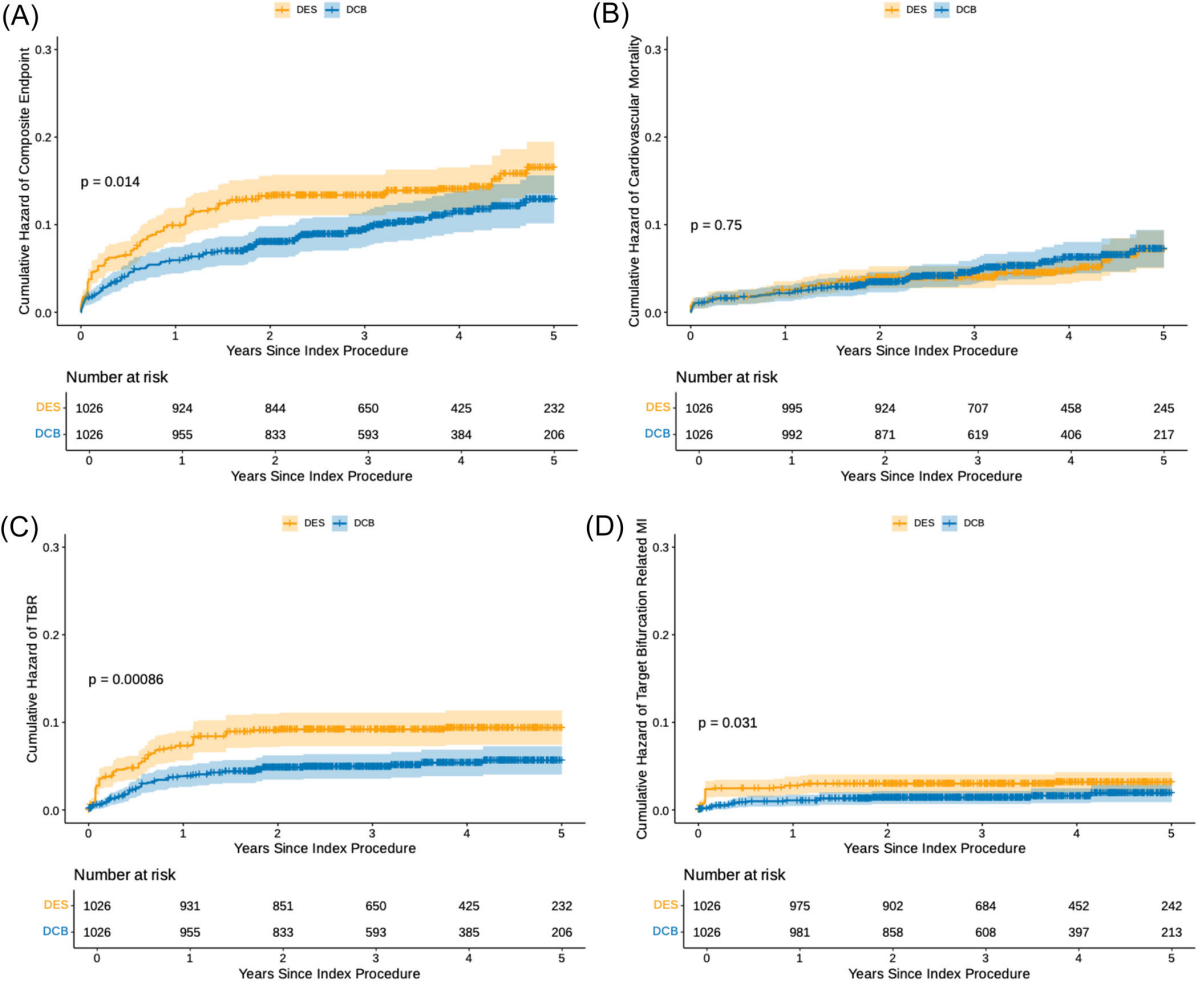
Cumulative hazard plot for propensity matched composite endpoint. (A) Shows the cumulative hazard plot for the propensity‐matched composite endpoint. (B) Shows the cumulative hazard plot for matched cardiovascular mortality. (C) The cumulative hazard plot for matched target lesion revascularization. (D) The cumulative hazard plot for matched target lesion myocardial infarction. [Color figure can be viewed at wileyonlinelibrary.com]

A propensity score matched analysis showed a significant increase in occurrence of events in the composite endpoint in the DES group (142/1026 [14.0%] vs. 102/1026 [9.9%], respectively) (HR: 1.39 [1.08−1.79], *p* = 0.01) up to 5 years (Figure 2, Panel A).

This increase in events in the DES arm was largely driven by increased TBR in the DES group (91/1026 [8.9] vs. 51/1026 [5.0%]) (HR: 1.79 [1.27−2.50], *p* < 0.001) (Figure 2, Panel C).

Furthermore, there was a significant difference in TB‐MI between the two groups (31/1026 [3.0%] vs. 16/1026 [1.6%] (HR: 1.92 [1.05−3.57], *p* < 0.03) (Figure 2, Panel D). The cause of spontaneous target bifurcation‐related MI is shown in Supporting Information S1: Table 5. There was no significant difference between the two groups with regard to cardiovascular mortality (54/1026 [5.3] vs. 54/1026 [5.3%]) (HR: 0.94 [0.65−1.37], *p* = 0.75) (Figure 2, Panel B).

A multivariate analysis (Table 3) for both the composite endpoint and all individual components of the composite endpoint is shown below.

**Table 3:** Multivariate analysis for the propensity‐matched cohort for the composite endpoint, target lesion revascularization, TL‐MI, and cardiovascular death.

	Composite endpoint		TBR		TB‐MI		CD	
Variable	Hazard ratio (CI)	*p* value	Hazard ratio (CI)	*p* value	Hazard ratio (CI)	*p* value	Hazard ratio (CI)	*p* value
DES (DCB)	1.43 (1.10−1.85)	**0.007**	1.82 (1.28−2.56)	**< 0.01**	0.59 (0.32−1.10)	0.09	0.98 (0.67−1.45)	0.93
Age	1.01 (1.00−1.02)	0.11	1.03 (1.01−1.04)	**0.002**	0.98 (0.95−1.01)	0.15	1.07 (1.05−1.09)	**< 0.001**
Sex (female)	0.92 (0.65−1.30)	0.63	0.68 (0.41−1.13)	0.14	0.63 (0.24−1.63)	0.34	1.03 (0.63−1.69)	0.92
Presentation								
Stable angina	—		—		—		—	** —**
ACS	2.02 (1.42−2.88)	**0.001**	1.70 (1.13−2.61)	**0.01**	1.49 (0.71−3.14)	0.29	2.13 (1.21−3.78)	0.009
STEMI	2.49 (1.71−3.63)	**< 0.001**	1.09 (0.66−1.81)	0.74	1.07 (0.45−2.51)	0.88	4.77 (2.73−8.34)	< 0.001
True bifurcation	1.47 (1.11−1.95)	**0.008**	1.63 (1.13−2.34)	**0.01**	3.13 (1.67−5.85)	**< 0.001**	1.16 (0.76−1.78)	0.49
Previous MI	1.07 (0.76−1.55)	0.66	1.17 (0.73−1.86)	0.52	2.05 (1.00−4.19)	**0.04**	—	—
Previous PCI	1.00 (0.68−1.48)	0.98	0.69 (0.42−1.15)	0.16	0.96 (0.43−2.17)	0.93	—	—
AF	2.17 (1.50−3.15)	**< 0.001**	2.07 (1.26−3.38)	**0.004**	5.65 (2.87−11.1)	**< 0.001**	—	—
Heavily calcified	2.04 (1.50−2.79)	**< 0.001**	2.82 (1.89−4.23)	**< 0.001**	1.40 (0.68−2.88)	0.36	1.52 (0.95−2.42)	0.07
Diffuse disease	0.84 (0.62−1.14)	0.27	0.91 (0.62−1.33)	0.62	0.57 (0.29−1.13)	0.11	0.77 (0.48−1.23)	0.28
Vessel diameter	1.08 (0.80−1.47)	0.61	0.83 (0.56−1.22)	0.34	0.69 (0.35−1.36)	0.28	1.47 (0.95−2.29)	0.08
Treated lesion length	1.01 (1.00−1.03)	**0.01**	1.00 (0.99−1.02)	0.86	1.02 (1.00−1.05)	0.06	1.03 (1.01−1.05)	**< 0.001**
Tortuosity	0.95 (0.68−1.33)	0.76	0.88 (0.57−1.35)	0.55	1.28 (0.63−2.60)	0.50	1.18 (0.71−1.05)	0.52

*Note:* Bold values indicate Statistically significant at *p* < 0.05.

Abbreviations: CD = cardiovascular death, CI = confidence interval, TB‐MI = target bifurcation‐related myocardial infarction, TBR = target bifurcation revascularization.

We conducted a sensitivity analysis of matching without replacement which highlights the appropriateness of matching with replacement as demonstrated in Supporting Information S1: Table 9 and Figure 2.

Finally, of the 63 patients treated with a hybrid DES/DCB strategy (DES MV and DCB SB), there was a total of 3.2.% (2/63) composite endpoint events, driven by target lesion revascularization with no occurrence of cardiovascular mortality (Central Figure 1).

**Central Figure 1:**
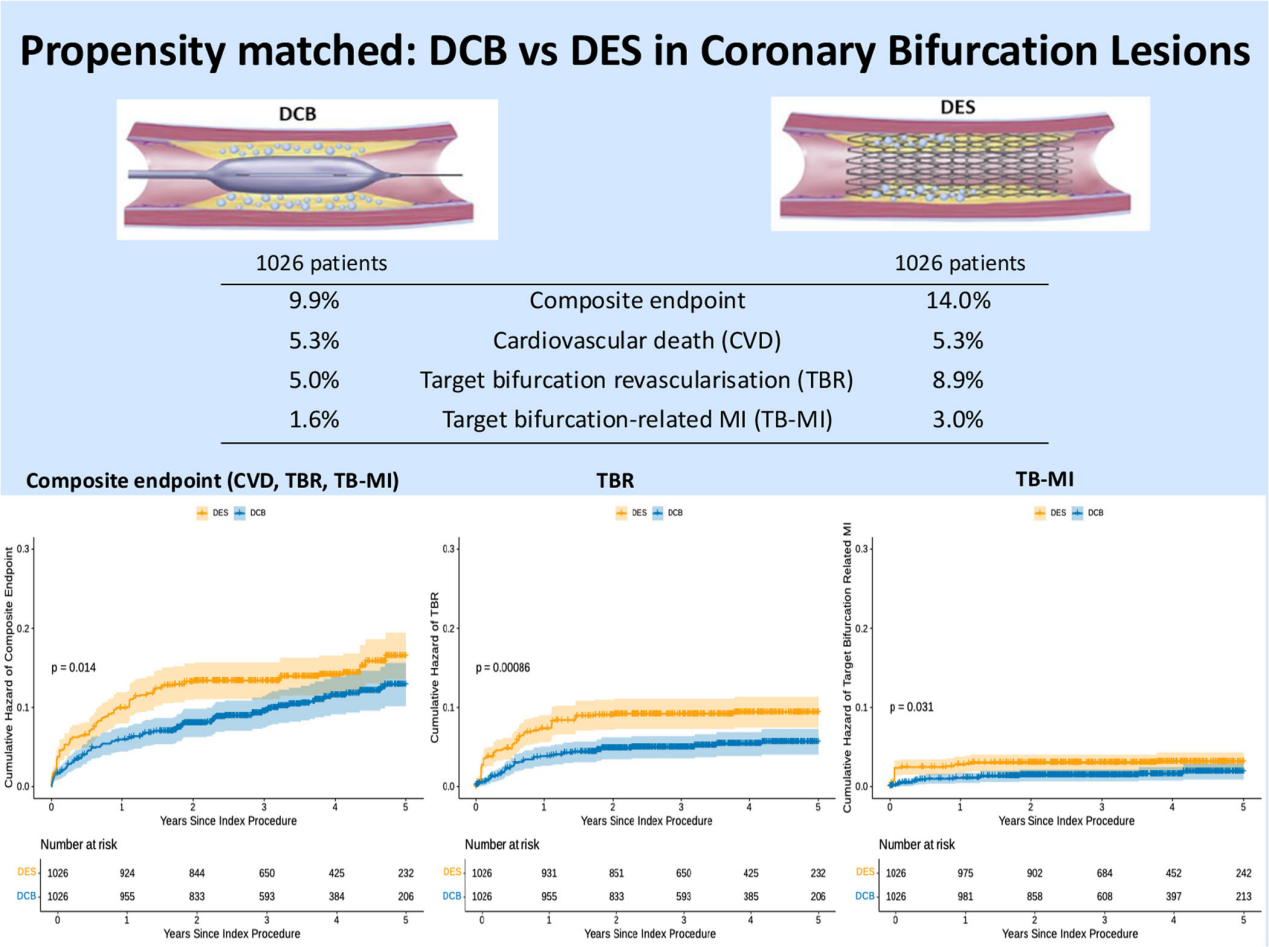
This is a propensity‐matched analysis comparing DCB‐only with DES‐only PCI for coronary bifurcation lesions including 2052 propensity‐matched patients. It shows a significant increase in bifurcation‐oriented composite endpoint (BOCE), target bifurcation revascularization, and target bifurcation‐related myocardial infarction with DES compared with DCB‐only PCI. [Color figure can be viewed at wileyonlinelibrary.com]

## 4 Discussion

To our knowledge, this is the largest cohort analysis comparing a DCB only approach with 2nd generation DES only as a treatment strategy for CBL, including 2113 patients, with 2052 patients included in the propensity‐matched analysis. We report that in our propensity‐matched population, which adjusted for higher lesion complexity seen in the DCB group, a significant reduction in the primary composite endpoint when treating a bifurcation lesion with a DCB compared with a DES was observed. This was largely due to a reduction in clinical TBR and TB‐MI.

Coronary bifurcation angioplasty remains associated with increased procedural complexity, peri‐procedural complications [22], and poorer long‐term clinical outcomes [23] than non‐bifurcation PCI, despite advances in techniques and technology. It is increasingly clear that a provisional strategy should be adopted where possible, although in the case of distal LMS bifurcation lesions, the evidence remains more ambiguous [24, 25]. The DEFINITION II trial has provided clear parameters for when a 2‐stent strategy should be adopted for complex bifurcation lesions [26], as endorsed by consensus documents [6, 27]. Recent results from long‐term follow‐up from EBC‐II go further to advocate a provisional strategy even in true bifurcations [28]. This is reflected in our practice and thus our data, with a two‐stent strategy accounting for only 4% of all bifurcations treated with DES. Our rates of two‐stent strategy appear lower than other bifurcation registries (17.1% in the COBIS III Registry [29]. However, this analysis accounts for any bifurcation lesion. There was no quantification of the significance of the side branch as seen in prospective bifurcation studies. In combined data from COBIS and BIFURCAT registries, 36.9% of true bifurcations had a two‐stent strategy [30], whereas in our study, 29.7% of true bifurcation lesions had a two‐stent strategy, suggesting there is no such discrepancy in two‐stent use for complex lesions. Furthermore, we hypothesize an institutional uptake of DCB use in the more complex bifurcation lesions over time, reflected by the higher true bifurcation lesions seen in the unmatched DCB group.

DCB angioplasty can further simplify a bifurcation procedure by reducing anatomical change at the carina and, most encouragingly, DCB to the main vessel facilitates lumen gain in the side branch [31]. A recent cost‐effectiveness analysis suggests no increased cost with this treatment strategy [32]. The recent DCB‐BIF study showed a benefit to DCB to the SB after a provisional MV strategy when compared to a noncompliant balloon [33]. However, the data is lacking for a DCB only strategy in bifurcation PCI with the largest registry to date reporting DCB outcomes for a main vessel strategy included 127 lesions treated with a 9‐month follow‐up [34]. This was limited by a high bailout stent rate (45%) and reported MACE at 9 months of 6.1%. Whilst the recent REC‐CAGEFREE I study failed to meet non‐inferiority when comparing DCB to DES in all vessel sizes, this excluded complex CBL [35].

We report the largest registry analysis for DCB angioplasty in bifurcation lesions, but also report the most relevant hard clinical endpoints appropriate for bifurcation device trials [17], including left main stem PCI and all clinical presentations, showing that DCB may be a safe and effective alternative treatment strategy to DES for bifurcation lesions. Propensity matching was undertaken in this cohort, as the DCB group had a significantly higher rate of true bifurcation lesions (30.0% compared to 20.5% respectively), as well as lesion complexity captured by vessel tortuosity, diffuse disease, and calcification. When adjusting for these variables, DCB use was associated with a reduction in the composite endpoint of cardiac death, TBR, and target bifurcation‐related MI. The importance of these clinically driven TBRs (as opposed to angiographically driven events in a number of the RCTs with angiographic follow‐up) is more relevant to the patient. The TBR rates reported in the DES group at 8.9% are very similar to the provisional strategy group in the 3‐year follow‐up from the Nordic study (6.2% vs. 6.1%) [36], with the COBIS registry reporting a 10.5% TLR rate in a one‐stent strategy (median follow‐up of 20 months) [37].

Finally, in the propensity‐matched group, we have shown a shorter procedure time, suggesting that DCBs provide a simpler approach. However, all of these findings need to be interpreted with caution in light of the significant limitations outlined below.

### 4.1 Selection and Approach of DCB Angioplasty

In our center, about half of our consultant operators adopt a DCB only strategy to most PCI. Our results suggest a trend to more complex lesions being treated with a DCB only approach (heavy calcification, diffuse disease, more tortuosity, and more true bifurcations in the unmatched DCB arm). This would suggest a selection bias favoring the DES cohort.

Our approach to a DCB‐only bifurcation PCI is a main vessel strategy only for most lesions, relying on the proven ostial SB LLG [38]. We only treat the side branch if there is a significant length of disease that would be viewed as requiring PCI independent of the MB disease. We do not routinely undertake kissing balloon angioplasty. Sequential DCB delivery is usually adequate when treating both branches, generally the side branch first (a complex 2‐lesion DCB‐only bifurcation stepwise approach would be sequential lesion prep main, lesion prep side, DCB side, DCB main).

### 4.2 Limitations

As a non‐randomized cohort study, this leads to inherent bias in the study design. We have sought to ameliorate this by including all consecutive patients who underwent bifurcation PCI with either a DES or DCB only in the study timeframe. Whilst patients with missing data were excluded from the analysis, this was only four patients in each group. Although this is a single‐center analysis, our center treats a large population of patients in excess of one million people. Our center was performing the highest rate of DCB only angioplasty in the United Kingdom at the time of this analysis [39]. Our results may not be reproducible in centers less experienced in DCB angioplasty currently, reflecting the learning curve associated with any interventional technique, which may impact on external validity of these. Our center has developed expertise in the use of DCB only angioplasty over a number of years and further experience in DCB only PCI may be required before operators undertake a DCB only approach to complex bifurcation lesions. The volume of DCB/DES bifurcation by year is shown in Supporting Information S1: Table 6.

By undertaking a propensity‐matched analysis for our cohort, we have sought to adjust at baseline for the clinically significant differences between the two groups. We cannot, however, in this retrospective analysis, identify intended bifurcation strategy for all patients and there may be patients with an initial intended DCB strategy that subsequently had DES implantation due to a suboptimal result after lesion preparation. Whilst the bailout to stent rate during this timeframe in our center after DCB was 3.8%, we cannot determine patients that had bailout stenting after lesion preparation. Our results therefore represent in the DCB only group a successful intended DCB approach, whilst the DES arm includes both those intended DES and those bailout stenting after failed lesion preparation intended for DCB.

Whilst this is a retrospective analysis, all patients are prospectively entered into our clinical database. By obtaining national database follow‐up, we have ensured completeness of follow‐up (accounting for the 61 patients [2.9%]) who have been excluded from analysis as these patients have opted out from HES data collection). As TBR is a component of the composite endpoint, there may be referrer bias for offering revascularization given operators are aware of the treatment strategy.

Our rates of intravascular imaging were low. This may reflect the historical time‐frame of the data collection, with BCIS data showing IVUS use in PCI in 2015 was < 10% in most centers. [40] There is limited evidence currently of the benefit of intravascular imaging in a planned DCB only strategy. In light of recent data [41], the importance of intravascular imaging when using DES for complex CBL is paramount and may reduce generalizability of the outcomes to contemporary practice. Whilst our data may therefore seem less relevant than intended, this is the only route to adequately sized cohort studies with follow‐up data extending far enough to reveal the importance of the ongoing DES attrition rate only recently appreciated (due to the shorter term follow‐up of many DES RCTs). We also acknowledge the lack of QCA data, but emphasize that this is a very large retrospective cohort study with an adequate clinical event rate (thereby perhaps negating the need for angiographic surrogate endpoints with their own associated limitations).

Lastly, we also note the low rates of completed 2‐lesion (i.e., more complex PCI) techniques. We believe that our adoption of a DCB only approach has allowed us to simplify many complex bifurcations (shown by the low rate of 2‐lesion approach even in the true bifurcation group) in both cohorts, as we believe in a minimalist safe approach to such potentially complex procedures.

## 5 Conclusion

This is the first cohort analysis comparing DCB and 2nd generation DES as a main treatment strategy for CBL reporting 2113 CBL, with 2056 patients in a propensity matched analysis reporting a significant reduction in composite endpoint events associated with DCB use compared to the DES arm. Our work shows the potential safety and efficacy of a DCB only approach.

This signifies an equipoise in the percutaneous treatment of CBL and justifies a randomized control study to investigate the reduction in event rate associated with DCB seen in this registry. Our results need to be interpreted with caution, particularly when considering the significant selection bias that exists in a non‐randomized retrospective analysis and the potential lack of generalizability given the expertise of our center with DCB only angioplasty. Hence, we would suggest these results, particularly in light of the recent REC‐CAGEFREE II trial, are hypothesis‐generating. However, it remains a very important step in investigating the role of a DCB only approach to a CBL and indicates the need for a well‐designed randomized controlled trial.

## SPARTAN‐Norfolk Consortium Members

Tim Gilbert, Clint Maart, Paul Richardson, Trevor Wistow, Chris Sawh, and Sulfi Sreekumar (Department of Cardiology, Norfolk and Norwich University Hospital). Johannes Reinhold and Alisdair Ryding (Department of Cardiology, Norfolk and Norwich University Hospita and Department of Medicine, University of East Anglia).

## Conflicts of Interest

Natasha Corballis and Vasiliki Tsampasian receive NIHR doctoral research fellowship funding. U. Bhalraam is an NIHR funded academic clinical fellow. Vassilios Vassiliou reports speaker fees from Sanofi and Daichii‐Sankyo and received grants for investigator‐initiated research from B Braun and Medtonic. Simon Eccleshall received speaker fees and acts as a consultant for B Braun, Medtronic and MedAlliance, and received grants for investigator‐initiated research from B Braun. Dr Ioannis Merinopoulos received a research grant from Cordis. The other authors declare no conflicts of interest.

## Supporting information


**Supplemental Figure 1:** Study Flow Diagram. **Supplemental Figure 2:** Sensitivity analysis showing propensity matching without replacement. **Supplemental Table 1:** Patient Characteristics for the whole cohort. **Supplemental Table 2:** Characteristics and binary outcomes of matched and unmatched patients for propensity matched analysis. **Supplemental Table 3:** Baseline Lesion/Procedural Characteristics. **Supplemental Table 4:** Procedural outcomes/complications for whole cohort. **Supplemental Table 5:** Cause of spontaneous target bifurcation‐related MI for the whole cohort. **Supplemental Table 6:** Number of DCB/DES bifurcation procedures per year. **Supplemental Table 7:** Univariable cox regression analysis for the unmatched primary composite endpoint. **Supplemental Table 8:** Multivariate cox regression analysis for the unmatched composite endpoint. **Supplemental Table 9:** Sensitivity analysis Table 1 showing matching without replacement baseline patient, angiographic and procedural characteristics.

## References

[1] J. F. Lassen , N. R. Holm , G. Stankovic , et al., “Percutaneous Coronary Intervention for Coronary Bifurcation Disease: Consensus From the First 10 Years of the European Bifurcation Club Meetings,” EuroIntervention 10, no. 5 (2014): 545–560, 10.4244/EIJV10I5A97. 25256198

[2] J. Al Suwaidi , W. Yeh , H. A. Cohen , K. M. Detre , D. O. Williams , and D. R. Holmes , “Immediate and One‐Year Outcome in Patients With Coronary Bifurcation Lesions in the Modern Era (NHLBI Dynamic Registry),” American Journal of Cardiology 87, no. 10 (2001): 1139–1144, 10.1016/s0002-9149(01)01482-5. 11356386

[3] C. Collet , T. Mizukami , and M. J. Grundeken , “Contemporary Techniques in Percutaneous Coronary Intervention for Bifurcation Lesions,” Expert Review of Cardiovascular Therapy 16, no. 10 (2018): 725–734, 10.1080/14779072.2018.1523717. 30221565

[4] K. H. Choi , F. Bruno , Y.‐K. Cho , et al., “Comparison of Outcomes Between 1‐Stent and 2‐Stent Techniques for Medina Classification 0.0.1 Coronary Bifurcation Lesions,” JACC: Cardiovascular Interventions 16, no. 17 (2023), 10.1016/J.JCIN.2023.06.013. 37565964

[5] M. Niemelä , K. Kervinen , A. Erglis , et al., “Randomized Comparison of Final Kissing Balloon Dilatation Versus No Final Kissing Balloon Dilatation in Patients With Coronary Bifurcation Lesions Treated With Main Vessel Stenting,” Circulation 123, no. 1 (2011): 79–86, 10.1161/CIRCULATIONAHA.110.966879. 21173348

[6] R. Albiero , F. Burzotta , J. L. Lassen , et al., “Treatment of Coronary Bifurcation Lesions, Part I: Implanting The First Stent in the Provisional Pathway. The 16th Expert Consensus Document of the European Bifurcation Club,” EuroIntervention 18, no. 5 (2022): e362–e376, 10.4244/EIJ-D-22-00165. 35570748 PMC10259243

[7] T. J. Ford , P. McCartney , D. Corcoran , et al., “Single‐ Versus 2‐Stent Strategies for Coronary Bifurcation Lesions: A Systematic Review and Meta‐Analysis of Randomized Trials With Long‐Term Follow‐up,” Journal of the American Heart Association 7, no. 11 (2018): 8730, 10.1161/JAHA.118.008730. PMC6015365 29802145

[8] J. R. L. Mínguez , J. M. N. Asensio , L. J. D. Vecino , et al., “A Prospective Randomised Study of the Paclitaxel‐Coated Balloon Catheter in Bifurcated Coronary Lesions (Babilon Trial): 24‐Month Clinical and Angiographic Results,” EuroIntervention 10, no. 1 (2014): 50–57, 10.4244/EIJV10I1A10. 24832638

[9] J. A. Herrador , J. C. Fernandez , M. Guzman , and V. Aragon , “Drug‐Eluting vs. Conventional Balloon for Side Branch Dilation in Coronary Bifurcations Treated by Provisional T Stenting,” Journal of Interventional Cardiology 26, no. 5 (2013): 454–462, 10.1111/joic.12061. 24106744

[10] F. X. Kleber , H. Rittger , J. Ludwig , et al., “Drug Eluting Balloons as Stand Alone Procedure for Coronary Bifurcational Lesions: Results of the Randomized Multicenter PEPCAD‐BIF Trial,” Clinical Research in Cardiology: Official Journal of the German Cardiac Society 105, no. 7 (2016): 613–621, 10.1007/s00392-015-0957-6. 26768146

[11] H. Liu , H. Tao , X. Han , et al., “Improved Outcomes of Combined Main Branch Stenting and Side Branch Drug‐Coated Balloon versus Two‐Stent Strategy in Patients With Left Main Bifurcation Lesions,” Journal of Interventional Cardiology 2022 (2022): 1–7, 10.1155/2022/8250057. PMC8767379 35095348

[12] R. V. Jeger , A. Farah , M.‐A. Ohlow , et al., “Long‐Term Efficacy and Safety of Drug‐Coated Balloons Versus Drug‐Eluting Stents for Small Coronary Artery Disease (BASKET‐SMALL 2): 3‐Year Follow‐Up of a Randomised, Non‐Inferiority Trial,” Lancet (London, England) 396, no. 10261 (2020): 1504–1510, 10.1016/S0140-6736(20)32173-5. 33091360

[13] T. T. Rissanen , S. Uskela , J. Eränen , et al., “Drug‐Coated Balloon for Treatment of De‐Novo Coronary Artery Lesions in Patients With High Bleeding Risk (DEBUT): A Single‐Blind, Randomised, Non‐Inferiority Trial,” Lancet 394, no. 10194 (2019): 230–239, 10.1016/S0140-6736(19)31126-2. 31204115

[14] I. Merinopoulos , T. Gunawardena , N. Corballis , et al., “Paclitaxel Drug‐Coated Balloon‐Only Angioplasty for De Novo Coronary Artery Disease in Elective Clinical Practice,” Clinical Research in Cardiology: Official Journal of the German Cardiac Society 112 (2022): 1186–1193, 10.1007/s00392-022-02106-y. 36104455 PMC10449668

[15] I. Merinopoulos , T. Gunawardena , N. Corballis , et al., “Assessment of Paclitaxel Drug‐Coated Balloon Only Angioplasty in STEMI,” JACC: Cardiovascular Interventions 16 (2023): 7, 10.1016/J.JCIN.2023.01.380. 37045498

[16] T. D. Gunawardena , N. Corballis , I. Merinopoulos , et al., “Drug‐Coated Balloon vs. Drug‐Eluting Stents for De Novo Unprotected Left Main Stem Disease: The SPARTAN‐LMS Study,” Journal of Cardiovascular Development and Disease 10, no. 2 (2023): 84, 10.3390/jcdd10020084. 36826580 PMC9963161

[17] M. Lunardi , Y. Louvard , M. Thierry Lefèvre , et al., “Definitions and Standardized Endpoints for Treatment of Coronary Bifurcations,” Journal of the American College of Cardiology 80 (2022): 185, 10.1016/J.JACC.2022.04.024. 35597684

[18] R. V. Jeger , S. Eccleshall , W. A. Wan Ahmad , et al., “Drug‐Coated Balloons for Coronary Artery Disease: Third Report of the International DCB Consensus Group,” JACC: Cardiovascular Interventions 13, no. 12 (2020): 1391–1402, 10.1016/j.jcin.2020.02.043. 32473887

[19] H. M. Garcia‐Garcia , E. P. McFadden , A. Farb , et al., “Standardized End Point Definitions for Coronary Intervention Trials: The Academic Research Consortium‐2 Consensus Document,” Circulation 137, no. 24 (2018): 2635–2650, 10.1161/CIRCULATIONAHA.117.029289. 29891620

[20] T. Gilbert , J. Neuburger , J. Kraindler , et al., “Development and Validation of a Hospital Frailty Risk Score Focusing on Older People in Acute Care Settings Using Electronic Hospital Records: An Observational Study,” Lancet (London, England) 391, no. 10132 (2018): 1775–1782, 10.1016/S0140-6736(18)30668-8. 29706364 PMC5946808

[21] D. E. Ho , K. Imai , G. King , and E. A. Stuart , “MatchIt: Nonparametric Preprocessing for Parametric Causal Inference,” Journal of Statistical Software 42, no. 8 (2011): 1–28, 10.18637/jss.v042.i08.

[22] I. Sheiban , Z. Ge , J. Kan , et al., “Association of Peri‐Procedural Myocardial Infarction With Mortality After Stenting True Coronary Bifurcation Lesions: A Pooled Individual Participant Data Analysis From Four Randomized Controlled Trials,” Catheterization and Cardiovascular Interventions 99, no. 3 (2022): 617–626, 10.1002/ccd.29946. 34494355

[23] J. M. Lee , S. H. Lee , J. Kim , et al., “Ten‐Year Trends in Coronary Bifurcation Percutaneous Coronary Intervention: Prognostic Effects of Patient and Lesion Characteristics, Devices, and Techniques,” Journal of the American Heart Association 10, no. 18 (2021): e021632, 10.1161/JAHA.121.021632. 34514841 PMC8649555

[24] D. Hildick‐Smith , M. Egred , A. Banning , et al., “The European Bifurcation Club Left Main Coronary Stent study: A Randomized Comparison of Stepwise Provisional vs. Systematic Dual Stenting Strategies (EBC MAIN),” European Heart Journal 42, no. 37 (2021): 3829–3839, 10.1093/eurheartj/ehab283. 34002215

[25] X. Chen , X. Li , J.‐J. Zhang , et al., “3‐Year Outcomes of the DKCRUSH‐V Trial Comparing DK Crush With Provisional Stenting for Left Main Bifurcation Lesions,” JACC: Cardiovascular Interventions 12, no. 19 (2019): 1927–1937, 10.1016/J.JCIN.2019.04.056. 31521645

[26] J.‐J. Zhang , F. Ye , K. Xu , et al., “Multicentre, Randomized Comparison of Two‐Stent and Provisional Stenting Techniques in Patients With Complex Coronary Bifurcation Lesions: The DEFINITION II trial,” European Heart Journal 41, no. 27 (2020): 2523–2536, 10.1093/eurheartj/ehaa543. 32588060

[27] J. L. Lassen , R. Albiero , T. J. Johnson , et al., “Treatment of Coronary Bifurcation Lesions, Part II: Implanting Two Stents. The 16th Expert Consensus Document of the European Bifurcation Club,” EuroIntervention 18, no. 6 (2022): 457–470, 10.4244/EIJ-D-22-00166. 35570753 PMC11064682

[28] S. Arunothayaraj , M. W. Behan , T. Lefèvre , et al., “Stepwise Provisional Versus Systematic Culotte for Stenting of True Coronary Bifurcation Lesions: Five‐Year Follow‐Up of the Multicentre Randomised EBC TWO Trial,” EuroIntervention 19, no. 4 (2023): e297–e304, 10.4244/EIJ-D-23-00211. 37946522 PMC10333921

[29] J. Kang , J.‐K. Han , H.‐M. Yang , et al., “Comparison of 2‐Stenting Strategies Depending on Sequence or Technique for Bifurcation Lesions in the Second‐Generation Drug‐Eluting Stent Era: Analysis From the COBIS (Coronary Bifurcation Stenting) III Registry,” Circulation Journal 85, no. 11 (2021): 1944–1955, 10.1253/circj.CJ-20-0999. 34078776

[30] J. H. Kim , L. Franchin , S. J. Hong , et al., “Two‐Year Clinical Outcomes After Coronary Bifurcation Stenting in Older Patients From Korea and Italy,” Frontiers in Cardiovascular Medicine 10 (2023): 1106594, 10.3389/fcvm.2023.1106594. 37034327 PMC10076885

[31] A. Y. Her , S. H. Ann , G. B. Singh , et al., “Serial Morphological Changes of Side‐Branch Ostium After Paclitaxel‐Coated Balloon Treatment of De Novo Coronary Lesions of Main Vessels,” Yonsei Medical Journal 57, no. 3 (2016): 606–613, 10.3349/ymj.2016.57.3.606. 26996558 PMC4800348

[32] I. Merinopoulos , T. Gunawardena , N. Corballis , et al., “Cost Effectiveness Analysis of Drug Coated Balloon Only Angioplasty for De Novo Coronary Artery Disease,” Catheterization and Cardiovascular Interventions 102, no. 6 (2023): 987–996, 10.1002/ccd.30878. 37925618

[33] X. Gao , N. Tian , J. Kan , et al., “Drug‐Coated Balloon Angioplasty of the Side Branch During Provisional Stenting: The Multicenter Randomized DCB‐BIF Trial,” Journal of the American College of Cardiology 85 (2024): 1, 10.1016/J.JACC.2024.08.067. 39480378

[34] L. Bruch , M. Zadura , M. Waliszewski , et al., “Results From the International Drug Coated Balloon Registry for the Treatment of Bifurcations. Can a Bifurcation be Treated Without Stents?,” Journal of Interventional Cardiology 29, no. 4 (2016): 348–356, 10.1111/joic.12301. 27242273

[35] C. Gao , X. He , F. Ouyang , et al., “Drug‐Coated Balloon Angioplasty With Rescue Stenting Versus Intended Stenting for the Treatment of Patients With De Novo Coronary Artery Lesions (REC‐CAGEFREE I): An Open‐Label, Randomised, Non‐Inferiority Trial,” Lancet (London, England) 404, no. 10457 (2024): 1040–1050, 10.1016/S0140-6736(24)01594-0. 39236727

[36] K. Kervinen , M. Niemelä , H. Romppanen , et al., “Clinical Outcome After Crush Versus Culotte Stenting of Coronary Artery Bifurcation Lesions: The Nordic Stent Technique Study 36‐Month Follow‐Up Results,” JACC: Cardiovascular Interventions 6, no. 11 (2013): 1160–1165, 10.1016/J.JCIN.2013.06.009. 24262616

[37] Y.‐S. Koh , P.‐J. Kim , K. Chang , et al., “Long‐Term Clinical Outcomes of the One‐Stent Technique Versus the Two‐Stent Technique for Non‐Left Main True Coronary Bifurcation Disease in the Era of Drug‐Eluting Stents,” Journal of Interventional Cardiology 26, no. 3 (2013): 245–253, 10.1111/joic.12025. 23480867

[38] T. Fujimura , T. Okamura , H. Tateishi , et al., “Serial Changes in the Side‐Branch Ostial Area After Main‐Vessel Stenting With Kissing Balloon Inflation for Coronary Bifurcation Lesions, Assessed by 3D Optical Coherence Tomography,” European Heart Journal‐Cardiovascular Imaging 19, no. 10 (2018): 1117–1125, 10.1093/ehjci/jex213. 29069325

[39] P. Ludman , “BCIS National Audit. Adult Interventional Procedures.”

[40] P. Ludman , “BCIS Audit Data 2015,” 2015.

[41] N. R. Holm , L. N. Andreasen , O. Neghabat , et al., “OCT or Angiography Guidance for PCI in Complex Bifurcation Lesions,” New England Journal of Medicine 389, no. 16 (2023): 1477–1487, 10.1056/NEJMoa2307770. 37634149

