# Supplementary material for: A Comparison of a Drug Coated Balloon With Drug Eluting Stent Strategy for Treating Coronary Bifurcation Lesions
Source: Catheter Cardiovasc Interv. 2025 Oct 28;107(1):3–14. doi: 10.1002/ccd.70273 (PMC12775198; doi:10.1002/ccd.70273)
Supplement: Supplementary file 1 — Supplemental Figure 1: Study Flow Diagram. Supplemental Figure 2: Sensitivity analysis showing propensity matching without replacement. Supplemental Table 1: Patient Characteristics for the whole cohort. Supplemental Table 2: Characteristics and binary outcomes of matched and unmatched patients for propensity matched analysis. Supplemental Table 3: Baseline Lesion/Procedural Characteristics. Supplemental Table 4: Procedural outcomes/complications for whole cohort. Supplemental Table 5: Cause of spontaneous target bifurcation‐related MI for the whole cohort. Supplemental Table 6: Number of DCB/DES bifurcation procedures per year. Supplemental Table 7: Univariable cox regression analysis for the unmatched primary composite endpoint. Supplemental Table 8: Multivariate cox regression analysis for the unmatched composite endpoint. Supplemental Table 9: Sensitivity analysis Table 1 showing matching without replacement baseline patient, angiographic and procedural characteristics. [file CCD-107-3-s001.docx]

Supplemental Table 1: Patient Characteristics for the whole cohort

| Patient characteristics | DCB (n=1030) (n,%) | DES (n=1083) (n,%) |  | P value |
| --- | --- | --- | --- | --- |
| Female sex | 204 (20.0) | 239 (22.0) |  | 0.20 ^1^ |
| Age (median, IQR) | 68 (59-75) | 67 (58-74) |  | 0.09 ^2^ |
| Frailty   - Low - Intermediate - High | 1008 (98.0)  22 (2.1)  0 (0.0) | 1066 (98.0)  16 (1.5)  1 (<0.1) |  | 0.33 ^3^ |
| Hypertension | 505 (49) | 489 (45) |  | 0.07 ^1^ |
| Dyslipidaemia | 243 (24) | 265 (24) |  | 0.64 ^1^ |
| Previous CVE | 55 (5.3) | 44 (4.1) |  | 0.16 ^1^ |
| Peripheral vascular disease | 38 (3.7) | 31 (2.9) |  | 0.29 ^1^ |
| Previous MI | 253 (25) | 216 (20) |  | **0.01** ^1^ |
| Previous PCI | 261 (25) | 225 (21) |  | **0.01** ^1^ |
| Previous CABG | 55 (5.3) | 51 (4.7) |  | 0.51 ^1^ |
| COPD | 57 (5.5) | 52 (4.8) |  | 0.45 ^1^ |
| Family history of coronary disease | 177 (17) | 167 (15) |  | 0.27 ^1^ |
| Diabetic | 201 (20) | 211 (20) |  | 0.99 ^1^ |
| Current/ ex-smoker | 603 (60) | 653 (63) |  | 0.23 ^1^ |
| Clinical presentation   - Stable - ACS (unstable angina/ NSTEMI) - STEMI | 338 (37)  384 (37)  268 (26) | 446 (41)  335 (31)  302 (28) |  | **0.008 ^1^** |
| Creatinine | 83 (72-98) | 83 (71-97) |  | 0.68 ^2^ |

1= Pearson Chi-squared test, 2= Wilcoxon Rank Sum test, 3=Fisher’s exact test

CVE= cerebrovascular event, MI= myocardial infarction, PCI= percutaneous coronary intervention, CABG= coronary artery bypass grafting, COPD= chronic obstructive pulmonary disease, ACS= acute coronary syndrome.

Supplemental Table 2: Characteristics and binary outcomes of matched and unmatched patients for propensity matched analysis

| Characteristic | Matched, n=1565 | Unmatched, n=539 | p-value |
| --- | --- | --- | --- |
| DCB  DES  Age, Median (IQR) | 1026  527  68 (58-75) | 0  551  68 (58-74) | 0.23 |
| Sex (Female) | 312 (20) | 130 (24) | 0.08 |
| Presentation  STEMI  ACS  Stable angina | 416 (27)  568 (37)  569 (37) | 152 (28)  150 (27)  249 (45) | **0.01** |
| True bifurcation | 435 (28) | 89 (16) | **<0.001** |
| Previous MI | 366 (24) | 96 (17) | **0.003** |
| Previous PCI | 377 (24) | 104 (19) | **0.009** |
| Diabetes | 304 (19) | 107 (19) | 0.98 |
| AF | 117 (7.5) | 29 (5.3) | 0.07 |
| Creatinine | 83 (72-96) | 83 (70-96) | 0.76 |
| Heavy calcification | 430 (28) | 117 (21) | **0.003** |
| Diffuse disease | 483 (31) | 117 (22) | **<0.001** |
| Tortuosity | 304 (19) | 52 (9.6) | **<0.001** |
| Vessel treated  LMS  Non-LMS | 77 (5.0)  1476 (95) | 46 (8.3)  505 (92) | **0.004** |
| Composite endpoint | 169 (11) | 58 (11) | 0.89 |
| Cardiovascular mortality | 84 (5.4) | 24 (4.4) | 0.34 |
| Target bifurcation revascularisation | 91 (5.9) | 34 (6.2) | 0.79 |
| Target bifurcation myocardial infarction | 31 (2.0) | 18 (3.3) | 0.08 |

IQR= interquartile range, STEMI= ST elevation myocardial infarction, ACS= acute coronary syndrome, MI=myocardial infarction, PCI= percutaneous coronary intervention, AF= atrial fibrillation, LMS= left main stem, LAD= left anterior descending, Cx= circumflex, RCA= right coronary artery

Supplemental Table 3: Baseline Lesion/ Procedural Characteristics

| Lesion characteristics | DCB (n=1030) (n,%) | DES (n=1083) (n,%) |  | P value |
| --- | --- | --- | --- | --- |
| Site of bifurcation disease:   - LMS - LAD - Cx - RCA - Graft | 49 (4.8)  618 (60)  252 (24)  109 (11)  2 (0.2) | 74 (6.8)  687 (63)  232 (21)  88 (8.1)  2 (0.2) |  | **0.03 ^1^** |
| Bifurcation lesion characteristics: |  |  |  |  |
| True bifurcation | 309 (30.0) | 223 (20.5) |  | **<0.01 ^1^** |
| Type (Medina)   - 111 - 110 - 101 - 011 - 100 - 010 - 001 | 207 (20)  173 (17)  49 (4.8)  53 (5.1)  154 (15)  245 (24)  149 (14) | 152 (14)  207 (19)  30 (2.8)  41 (3.8)  222 (20)  342 (32)  89 (8.2) |  | **<0.01 ^1^** |
| Treatment strategy   - One vessel strategy - Two vessel strategy   Two stent strategy:  DK crush  Culotte  Tap  Shotgun  SKS | 922 (89.5)  108 (10.5) | 1041 (96.1)  42 (3.9)  25 (60.3)  8 (19.1)  3 (4.8)  4 (10.0)  2 (5.8) |  | **<0.01 ^1^** |
| Main vessel diameter, Median (IQR) | 3 (2.75-3.5) | 3.5 (3-3.75) |  | **<0.01 ^2^** |
| Treated side branch diameter, Median (IQR) | 2.7 (2.25-3) | 2.7 (2.25-3) |  | 0.66 ^2^ |
| Main vessel treated length | 20 (20-30) | 24 (18-32) |  | 0.08 ^2^ |
| Treated side branch length | 15 (15-20) | 15 (12-20) |  | **0.03** **^2^** |
| Heavy calcification | 291 (28) | 259 (24) |  | **0.02 ^1^** |
| Diffuse disease | 331 (32) | 273 (25) |  | **<0.01 ^1^** |
| Tortuosity | 216 (21) | 142 (13) |  | **<0.001 ^1^** |
| Coronary dissection after DCB  No dissection  Type A  Type B  Type C | 546 (53)  196 (19)  279 (27)  9 (1) | N/A |  |  |
| Pressure wire use | 118 (11) | 114 (11) |  | 0.49 ^1^ |
| Fluoroscopy time (mins), Median (IQR) | 12 (8-17) | 12 (9-19) |  | **0.035 ^2^** |
| Contrast used (mls), Median (IQR) | 130 (100-160) | 140 (100-170) |  | **0.005 ^2^** |

1= Pearson Chi-squared test, 2= Wilcoxon Rank Sum test

LMS= left main stem, LAD= left anterior descending artery, Cx= circumflex artery, RCA= right coronary artery.

Supplemental Table 4: Procedural outcomes/ complications for whole cohort

|  | DCB (1030) | DES (1083) |
| --- | --- | --- |
| No complication | 987 (95.8) | 1020 (94.2) |
| No reflow | 6 (0.6) | 9 (0.8) |
| Coronary perforation | 1 (0.1) | 1 (0.1) |
| Procedure induced shock | 1 (0.1) | 3 (0.3) |
| Side branch occlusion | 6 (0.6) | 16 (1.5) |
| Arrhythmia | 11 (1.1) | 19 (1.8) |
| Access site haematoma | 3 (0.3) | 0 (0.0) |
| Tamponade | 0 (0) | 1 (0.1) |
| CVE/TIA | 0 (0) | 1 (0.1) |
| Periprocedural MI | 0 | 2 (0.2) |

Supplemental Table 5: Cause of spontaneous target bifurcation-related MI for the whole cohort

| Reason for TB-MI | DCB (n=16)(% of TB-MI) | DES (n=31)(% of TB-MI) |
| --- | --- | --- |
| Restenosis  Thrombosis  SB related  Dissection | 10 (62.5)  2 (12.5)  0 (0.0)  4 (25.0) | 12 (39.4)  11 (36.4)  7 (21.2)  1 (3.0) |

Note: n=31 for DES as 2 TB-MI were periprocedural.

Supplmental Table 6: Number of DCB/DES bifurcation procedures per year

| Year of PCI | DCB | DES |
| --- | --- | --- |
| 2015 | 159 | 220 |
| 2016 | 200 | 197 |
| 2017 | 202 | 252 |
| 2018 | 261 | 220 |
| 2019 | 208 | 194 |

Supplemental Table 7: Univariable cox regression analysis for the unmatched primary composite endpoint

| Variable | Hazard ratio (CI) | P value |
| --- | --- | --- |
| Age | 1.02 (1.01-1.03) | **<0.001** |
| DES | 1.14 (0.88-1.48) | 0.33 |
| Female sex | 1.11 (0.81-1.51) | 0.53 |
| Presentation  STEMI  ACS  Stable angina | -  0.99 (0.73-1.34)  0.57 (0.41-0.80) | 0.94  **0.001** |
| True bifurcation | 1.72 (1.31-2.26) | **<0.001** |
| 2 stent strategy | 2.93 (1.57-5.45) | **<0.001** |
| Frailty | 1.12 (1.04-1.21) | **0.002** |
| Hypertension | 1.17 (0.90-1.51) | 0.24 |
| Dyslipidaemia | 0.87 (0.63-1.18) | 0.37 |
| Peripheral vascular disease | 1.40 (0.74-2.64) | 0.30 |
| CVE | 1.15 (0.64-2.05) | 0.64 |
| Previous MI | 1.20 (0.89-1.62) | 0.22 |
| Previous PCI | 1.04 (0.76-1.41) | 0.82 |
| Previous CABG | 2.21 (1.43-3.40) | **<0.001** |
| Heart failure | 2.83 (1.54-5.19) | **<0.001** |
| Family history CAD | 0.60 (0.39-0.90) | **0.014** |
| Asthma | 2.82 (1.89-4.22) | **<0.001** |
| COPD | 2.82 (1.89-4.22) | **<0.001** |
| Diabetes | 1.34 (0.99-1.82) | 0.059 |
| Smoking history | 1.06 (0.80-1.39) | 0.70 |
| Creatinine | 1.00 (1.00-1.01) | **<0.001** |
| Vessel treated:  LMS  LAD  Cx  RCA  Graft | -  0.49 (0.32-0.75)  0.45 (0.28-0.72)  0.32 (0.17-0.62)  1.29 (0.17-9.50) | **<0.001**  **0.001**  **<0.001**  0.80 |
| Multivessel PCI | 1.46 (1.07-2.00) | **0.017** |
| Heavily calcified | 2.36 (1.82-3.07) | **<0.001** |
| Diffuse disease | 1.19 (0.90-1.57) | 0.22 |
| Tortuosity | 1.52 (1.11-2.07) | **0.008** |
| Vessel diameter | 1.03 (0.86-1.24) | 0.75 |
| Treated lesion length | 1.01 (1.00-1.02) | 0.19 |

CAD= coronary artery disease. The remaining abbreviations as per tables 1 &2.

Supplemental Table 8: Multivariate cox regression analysis for the unmatched composite endpoint

| Variable | Hazard ratio (CI) | P value |
| --- | --- | --- |
| DCB/DES (DES) | 1.20 (0.91-1.58) | 0.20 |
| Presentation  STEMI  ACS  Stable angina | -  0.80 (0.57-1.12)  0.42 (0.28-0.61) | 0.20  **<0.001** |
| Heavy calcification | 2.02 (1.47-2.94) | **<0.001** |
| Contrast use (mls) | 1.00 (1.00-1.00 | 0.11 |
| True bifurcation | 1.43 (1.07-1.92) | **0.016** |
| Previous MI | 1.33 (0.96-1.84) | 0.084 |

Supplemental Table 9: Sensitivity analysis Table 1 showing matching without replacement baseline patient, angiographic and procedural characteristics

| **Baseline Characteristics** | **DES** N = 1,026 | **DCB** N = 1,026 | **p-value** |
| --- | --- | --- | --- |
| Age, Median (IQR) | 67 (58 – 74) | 68 (59 – 75) | 0.082*^1^* |
| Female | 223 (22) | 203 (20) | 0.28*^2^* |
| Presentation, n (%) |  |  | **0.039***^2^* |
| STEMI | 289 (28) | 267 (26) |  |
| ACS | 328 (32) | 383 (37) |  |
| Stable Angina | 409 (40) | 376 (37) |  |
| BIF_Medina_Classification, n (%) |  |  | **<0.001***^2^* |
| 1 | 146 (14) | 206 (20) |  |
| 2 | 196 (19) | 173 (17) |  |
| 3 | 29 (2.8) | 49 (4.8) |  |
| 4 | 41 (4.0) | 53 (5.2) |  |
| 5 | 203 (20) | 153 (15) |  |
| 6 | 324 (32) | 243 (24) |  |
| 7 | 87 (8.5) | 149 (15) |  |
| True_Bif, n (%) | 212 (21) | 306 (30) | **<0.001***^2^* |
| Not Frail | 1,011 (99) | 1,004 (98) |  |
| Moderately Frail | 14 (1.4) | 22 (2.1) |  |
| Severe Frailty | 1 (<0.1) | 0 (0) |  |
| Pressure_Wire, n (%) | 108 (11) | 118 (12) | 0.48*^2^* |
| Imaging, n (%) |  |  | 0.55*^2^* |
| IVUS | 48 (65) | 22 (71) |  |
| OCT | 26 (35) | 9 (29) |  |
| Dyslipidaemia | 246 (24) | 242 (24) | 0.84*^2^* |
| Hypertension | 461 (45) | 503 (49) | 0.063*^2^* |
| CVE | 41 (4.0) | 55 (5.4) | 0.14*^2^* |
| Previous MI | 207 (20) | 250 (24) | **0.023***^2^* |
| Previous PCI | 213 (21) | 260 (25) | **0.014***^2^* |
| AF | 60 (5.8) | 84 (8.2) | **0.038***^2^* |
| Previous CABG | 50 (4.9) | 55 (5.4) | 0.62*^2^* |
| Family history of CAD | 152 (15) | 177 (17) | 0.13*^2^* |
| COPD | 48 (4.7) | 57 (5.6) | 0.37*^2^* |
| Diabetes | 199 (19) | 199 (19) | >0.99*^2^* |
| Current/Ex Smoker | 615 (62) | 601 (60) | 0.33*^2^* |
| Creatinine, Median (IQR) | 83 (71 – 96) | 83 (71 – 97) | 0.43*^1^* |
| Vessel treated: |  |  | 0.11*^3^* |
| LMS | 58 (5.7) | 49 (4.8) |  |
| LAD | 655 (64) | 616 (60) |  |
| LCx | 231 (23) | 250 (24) |  |
| RCA | 81 (7.9) | 109 (11) |  |
| Graft | 1 (<0.1) | 2 (0.2) |  |
| Heavy calcification | 246 (24) | 289 (28) | **0.031***^2^* |
| Diffuse disease | 255 (25) | 329 (32) | **<0.001***^2^* |
| Tortuosity | 138 (13) | 215 (21) | **<0.001***^2^* |
| Main vessel diameter Median (IQR) | 3.50 (3.00 – 3.50) | 3.00 (2.75 – 3.50) | **<0.001***^1^* |
| Main vessel treated lesion length , Median (IQR) | 24 (18 – 32) | 20 (20 – 30) | 0.16*^1^* |
| Side branch treated length, Median (IQR) | 15 (12 – 20) | 15 (15 – 20) | **0.043***^1^* |

Supplemental Figure 1: Study Flow Diagram

Supplemental Figure 1 shows the study flow diagram of patients identified, excluded from the analysis and the final numbers included in the full and propensity matched analysis.

Supplmental Figure 2: Sensitivity analysis showing propensity matching without replacement.

Supplemental figure 2 shows cumulative hazard plots to 5 year follow-up for the composite endpoint and individual components when matching without replacement. Due to significant differences between the cohort and a very similar number of patients in each arm, this match was suboptimal as demonstrated in supplmental table 9.
